# Type 1 Diabetes Mellitus as a Model of Body Composition Transformation: Pathogenetic Unity of Combined Phenotypes

**DOI:** 10.3390/ijms27156852

**Published:** 2026-07-30

**Authors:** Regina Gizatullina, Karina Akhiiarova, Ildar Minniakhmetov, Rita Khusainova, Natalia Mokrysheva, Anton Tyurin

**Affiliations:** 1 Department of Internal Medicine, Bashkir State Medical University, 450008 Ufa, Russia; rina-sharipova@mail.ru (R.G.); liciadesu@gmail.com (K.A.); ritakh@mail.ru (R.K.); 2 Laboratory of Genomic Medicine, Endocrinology Research Centre, 117292 Moscow, Russia; minniakhmetov.ildar@endocrincentr.ru (I.M.); mokrisheva.natalia@endocrincentr.ru (N.M.)

**Keywords:** body composition, BC, type 1 diabetes mellitus, T1DM, sarcopenic obesity, osteosarcopenia, osteopenic obesity, osteosarcopenic obesity

## Abstract

The study of body composition (BC) is of increasing clinical and research relevance, as it provides a detailed characterization of metabolic status beyond traditional anthropometric indices such as body mass index (BMI). BC describes the quantitative ratio of fat mass (FM), lean mass (LM)—including skeletal muscle and internal organs—and bone mineral density (BMD). The pathogenetic mechanisms underlying specific BC phenotypes arise from a complex interplay of endocrine, metabolic, inflammatory, and genetic factors. Given the shared pathways involved in phenotype formation, the study of BC alterations across different diseases holds promise for predicting complications and identifying common therapeutic targets. This review aims to systematize current knowledge on combined BC phenotypes—sarcopenic obesity, osteosarcopenia, osteopenic obesity, and osteosarcopenic obesity—and to explore their pathogenetic unity, with particular emphasis on type 1 diabetes mellitus as a dynamic model of sequential phenotypic shifts.

## 1. Introduction

The study of body composition (BC) is gaining increasing relevance with each passing year. BC describes the quantitative ratio of the major tissue components of the organism: fat mass (FM), lean mass (LM), which includes skeletal muscle and internal organs, as well as bone mineral density (BMD). Assessment of BC allows one to move beyond the traditional use of body mass index (BMI) and obtain a detailed characterization of the patient’s metabolic status. A wide range of methods is employed for the quantitative assessment of BC, ranging from simple anthropometric measurements and bioelectrical impedance analysis (BIA) to highly precise instrumental technologies. Dual-energy X-ray absorptiometry (DXA) is currently the recognized clinical standard for BC assessment, enabling non-invasive measurement of fat mass, lean mass, and BMD [1]. Computed tomography (CT) and magnetic resonance imaging (MRI) provide the most accurate tissue differentiation at the organ level, including the ability to distinguish between visceral and subcutaneous adipose tissue; however, their use is limited by high cost and radiation exposure. BIA represents a more accessible alternative for routine clinical practice, although it is inferior to DXA in the accuracy of estimating fat and lean masses, particularly in the presence of fluid-electrolyte disturbances [2,3,4].

The pathogenetic mechanisms underlying the formation of a given BC phenotype result from a delicate balance at the intersection of endocrinology, cardiology, geriatrics, internal medicine in general, as well as genetics. Given the shared mechanisms of BC phenotype formation, the study of BC characteristics in various pathologies will allow prediction of possible complications and identification of common therapeutic targets.

BC alterations should be examined in two complementary perspectives. In the first, cross-nosological perspective, the same pathological body composition phenotype consistently develops across different diseases that share common pathogenetic pathways. In the second, dynamic perspective, in a single patient within a single nosology, different phenotypes successively replace one another as the disease progresses and under the influence of therapy. The present review is devoted to systematization of current data on combined phenotypes of BC alterations and their pathogenetic unity (Part I), as well as to the consideration of type 1 diabetes mellitus (T1DM) as a model of dynamic phenotypic shifts in body composition over time (Part II).

## 2. Part I. Combined Phenotypes of Body Composition: Epidemiology, Pathogenesis, and Clinical Significance

In clinical practice, isolated body composition phenotypes such as obesity, sarcopenia, and the combination of osteopenia and/or osteoporosis, along with their impact on patients’ quality of life, have traditionally been considered. However, accumulated evidence indicates that these phenotypes may share common pathogenetic pathways and, upon formation, exert synergistic negative effects on various organs and systems. The concept of combined body composition phenotypes, namely sarcopenic obesity, osteosarcopenia, osteopenic obesity, and osteosarcopenic obesity, has gained widespread recognition over the past two decades, and a growing body of literature continues to update the accumulated knowledge [5,6,7]. At the same time, several fundamentally important unresolved questions remain in this field, including the absence of universally accepted unified diagnostic criteria for each of the combined phenotypes, as well as the fragmentary nature of data on combined body composition phenotypes associated with specific nosological entities. It remains unclear which particular phenotype is characteristic of a given disease.

### 2.1. Normal-Weight Obesity as a Hidden Pathway to Combined Body Composition Phenotypes

Normal-weight obesity (NWO) is a condition in which individuals with a normal BMI (18.5–24.9 kg/m^2^) exhibit excess adipose tissue content (≥30% in women and ≥25% in men by DXA). The prevalence of NWO in the general population is approximately 10% [8,9]. Meanwhile, in terms of metabolic characteristics, individuals with NWO differ fundamentally from those with normal body composition: they exhibit chronic low-grade systemic inflammation, insulin resistance, dyslipidemia, and oxidative stress, comparable to those observed in overt obesity [10]. Critically, NWO is associated with a high risk of developing sarcopenia. In the clinical study by De Renzo et al., the presence of NWO increased the risk of sarcopenia 22-fold in men and 25-fold in women at the same BMI [11]. Concurrently, the pro-inflammatory profile of NWO may create conditions favoring accelerated decline in bone mineral density (BMD), potentially representing an early step toward osteosarcopenic obesity; direct longitudinal evidence linking NWO to incident osteosarcopenic obesity is still limited and warrants confirmation in prospective cohorts. Polymorphisms of pro-inflammatory cytokine genes, including interleukin-6 (IL-6), interleukin-1 receptor antagonist (IL-1Ra), interleukin-15 (IL-15), interleukin-15 receptor alpha subunit (IL-15Rα), and the *MTHFR* gene, play a role in genetic predisposition to NWO and associated sarcopenia. This indicates that NWO is not merely a stage of obesity but a distinct metabolic phenotype requiring an independent diagnostic and therapeutic strategy [10]. Thus, NWO represents a hidden entry point into the spectrum of combined pathological body composition phenotypes. Clinically, this implies that body composition screening using DXA or BIA is necessary even in patients with normal BMI—particularly in the presence of inflammatory diseases, physical inactivity, and nutritional disorders. As discussed in detail in Part II, T1DM provides a particularly illustrative example of this trajectory, since NWO-like adiposity is a characteristic intermediate phenotype that emerges after insulin therapy initiation and precedes the later shift toward sarcopenic and osteosarcopenic phenotypes.

### 2.2. Sarcopenic Obesity

Sarcopenic obesity (SO) is a pathological condition that meets the diagnostic criteria for both sarcopenia and obesity, as previously mentioned. Sarcopenia is diagnosed based on reduced muscle mass (appendicular lean mass < 7.0 kg/m^2^ in men and <5.5 kg/m^2^ in women by DXA), reduced muscle strength (handgrip strength < 27 kg in men and <16 kg in women), and/or reduced physical performance (gait speed < 0.8 m/s) in accordance with the EWGSOP2 2019 criteria [12]. According to population-based studies and meta-analyses, the global prevalence of sarcopenic obesity in older adults averages 11%. The incidence of the condition is significantly influenced by methodological and demographic factors. The highest rates, reaching 23%, have been recorded among patients aged over 75 years. The considerable variability in prevalence estimates is also attributable to the absence of unified diagnostic criteria, differences in the methods used for body composition assessment (DXA, BIA, anthropometry), and the characteristics of the studied populations [13,14,15]. At the same time, there is reason to believe that the actual and projected prevalence of sarcopenic obesity will steadily increase, which is directly driven by the global rise in the prevalence of obesity as its key component [16,17].

It should be emphasized that the EWGSOP2 criteria cited above were developed and validated predominantly in cohorts of older adults, and their absolute cut-off values for appendicular lean mass, handgrip strength, and gait speed are not directly transferable to children, adolescents, and young adults [18]. In pediatric and adolescent populations, sarcopenia and sarcopenic obesity are more commonly identified using percentile- or Z-score-based approaches referenced to age- and sex-matched healthy populations rather than fixed absolute thresholds. A systematic review applying such heterogeneous criteria across 18 pediatric studies reported a prevalence of sarcopenic obesity ranging from 5.66% to 69.7% in girls and from 7.2% to 81.3% in boys, underscoring the absence of a unified definition for younger age groups [18]. This methodological gap is particularly relevant for T1DM, which typically manifests in childhood, adolescence, or young adulthood: a recent systematic review focused specifically on sarcopenia in T1DM reported prevalence estimates ranging from 8% to 40%, depending on age, ethnicity, and the diagnostic criteria applied, and explicitly identified the development of age- and disease-specific diagnostic criteria as a priority for future research [19]. Reflecting this gap, studies in adult T1DM cohorts have used percentile-based thresholds derived from national reference populations rather than the EWGSOP2 absolute cut-offs, precisely because of the relatively young age of their participants [20]. These considerations should be borne in mind when interpreting the sarcopenia-related phenotypic shifts described for T1DM in Part II of this review.

#### 2.2.1. Pathogenetic Mechanisms of Sarcopenic Obesity

The pathogenesis of SO is determined by a complex interplay of genetic, inflammatory, and metabolic factors, in which obesity and sarcopenia mutually potentiate each other’s progression, forming a self-perpetuating pathological cascade [21]. One of the central mechanisms is chronic low-grade systemic inflammation: hypersecretion of tumor necrosis factor-alpha (TNF-α), IL-6, and interleukin-1 beta (IL-1β) by visceral adipose tissue activates the transcription factor nuclear factor kappa-B (NF-κB), triggers the ubiquitin-proteasome system (UPS) of proteolysis in skeletal muscle, and leads to caspase-mediated apoptosis of myocytes [22].

A significant contribution to the maintenance of the inflammatory milieu is made by NLRP3 (NLR Family Pyrin Domain Containing 3) inflammasome activation through damage-associated molecular patterns (DAMPs) such as adenosine triphosphate (ATP), advanced glycation end-products (AGEs), free fatty acids, and cholesterol crystals. Pathogen-associated molecular patterns (PAMPs), such as lipopolysaccharides that enter the systemic circulation due to intestinal barrier disruption in obesity, also contribute to low-grade inflammation by binding to Toll-like receptors (TLRs) on adipocytes and myocytes, triggering the assembly of the NLRP3-ASC-caspase-1 complex. Activated caspase-1 processes pro-interleukin-18 (pro-IL-18) and pro-interleukin-1 beta (pro-IL-1β) into their active forms, which further amplify the pro-inflammatory cascade and accelerate the degradation of sarcomeric proteins via the ubiquitin-proteasome system [23,24]. Intermuscular adipose tissue (IMAT) and intramyocellular lipid accumulation induce impairment of insulin signaling through activation of protein kinase C and accumulation of diacylglycerol and ceramides, resulting in reduced insulin-stimulated glucose uptake. The developing myosteatosis further impairs muscle contractile function through disruption of calcium homeostasis and reduced expression of myosin heavy chains [25].

Adipocyte hypertrophy in obesity induces adipose tissue hypoxia and endoplasmic reticulum stress (ER stress), leading to the release of the chemokine MCP-1 (monocyte chemoattractant protein-1) and adipocyte necrosis with the release of additional DAMPs—cholesterol crystals and free lipids. MCP-1-recruited monocytes differentiate into pro-inflammatory M1 macrophages, which produce TNF-α, IL-6, and IL-1β. Through the systemic circulation, these cytokines reach muscle tissue and activate signaling pathways of the ubiquitin-proteasome system and autophagy, i.e., the two major catabolic systems of muscle proteolysis [26].

Dysregulation of adipokines represents another key link in the pathogenesis. In obesity, adiponectin levels, which possess anti-inflammatory properties, are reduced, and leptin resistance develops. High leptin concentrations activate pro-inflammatory signaling cascades of janus kinase-2/signal transducer and activator of transcription-3 (JAK2/STAT3) and mitogen-activated protein kinase (MAPK) in muscle tissue, promoting the degradation of myofibrillar proteins. Reduced physical activity, resulting from excess body weight, closes the vicious circle: the deficit of mechanical loading suppresses myogenesis and enhances muscle protein catabolism [26].

#### 2.2.2. Sarcopenic Obesity in Specific Nosological Entities

It should be added that the variability in the prevalence of SO may also be attributable to insufficient attention to comorbid conditions predisposing to the formation of a given BC phenotype. At the same time, accumulating evidence indicates a consistent predisposition to the development of SO in certain nosological entities.

Type 2 diabetes mellitus (T2DM) is one of the most significant pathologies associated with SO: insulin resistance impairs anabolic PI3K/Akt/mTOR signaling in myocytes, whereas accumulation of intramuscular adipose tissue and advanced glycation end-products further suppresses muscle contractile function [27,28]. According to a systematic review and meta-analysis encompassing 20 studies, the prevalence of SO among patients with T2DM is approximately 27%, and its presence is associated with significantly higher risks of adverse outcomes [29]. NLRP3 inflammasome blockade may represent a promising direction for therapeutic intervention in this nosology [30]. Among diseases characterized by the development of SO, rheumatoid arthritis (RA) should also be considered. The prevalence of SO in RA, according to prospective studies with body composition assessment by DXA, ranges from 8.4% to 12.6%, which is 2–3 times higher than corresponding rates in the general population of comparable age [31]. The mechanism underlying the formation of this phenotype is driven by chronic hypersecretion of TNF-α and IL-6 from the synovium, which, upon entering the systemic circulation, activate NF-κB-dependent muscle catabolism; forced physical inactivity and corticosteroid therapy further contribute to visceral adipose tissue accumulation. Notably, these body composition alterations may be detectable even before clinically overt manifestations of RA [32]. Chronic obstructive pulmonary disease (COPD) is also associated with this phenotype: systemic inflammation with elevated circulating levels of TNF-α, IL-6, and C-reactive protein activates NF-κB-dependent proteolysis via the ubiquitin-proteasome system, whereas chronic hypoxemia suppresses protein synthesis through REDD1/AMPK-dependent inhibition of mTORC1, and glucocorticoids used during exacerbations further inhibit IGF-1/Akt signaling and stimulate myostatin expression, which is a negative regulator of muscle mass. Collectively, these mechanisms create conditions for progressive muscle loss with relative preservation or accumulation of adipose tissue [33,34,35]. The prevalence of SO in COPD, according to cross-sectional studies, is approximately 9.6%, while the overall frequency of sarcopenia in this patient population, based on a meta-analysis of 15 studies, reaches 29%, which significantly exceeds that in the population without obstructive pulmonary pathology [36,37].

### 2.3. Osteopenic Obesity

Osteopenic obesity (OPO) is a comorbid condition in which reduced BMD and obesity are simultaneously present in the same patient. This combination is characterized by a complex and often paradoxical pathogenetic interaction between adipose and bone tissues. It should be emphasized that the relationship between adipose tissue and bone metabolism is not unequivocally negative; for a long time, clinicians adhered to the concept of a protective role of obesity with respect to BMD, and this viewpoint has a biological rationale [38,39]. Increased body weight creates mechanical loading on the skeleton, stimulating osteoblastogenesis and bone remodeling [40]. At the same time, the protective effect of adipose tissue on bone is largely determined not so much by the quantity as by the type and topography of fat depots. Accumulated evidence convincingly demonstrates that subcutaneous adipose tissue is associated with higher BMD, whereas visceral adipose tissue exerts osteodestructive effects: hypersecretion of pro-inflammatory cytokines by visceral adipocytes activates bone resorption and suppresses osteoblast function [41]. Thus, the relationship between adipose tissue and bone is nonlinear and is determined by the balance between protective and destructive (visceral inflammation, insulin resistance) mechanisms. Systematic data on the prevalence of the specific combination of OPO remain limited due to the insufficient number of dedicated population-based studies.

#### Pathogenesis of Osteopenic Obesity

As noted earlier, obesity has traditionally been considered a protective factor with respect to BMD, primarily due to mechanical loading on the skeleton and increased peripheral conversion of androgens to estrogens in adipose tissue. However, current evidence indicates that it is visceral, rather than subcutaneous, obesity that is associated with reduced BMD [26,38]. This paradox is largely explained by the pro-inflammatory activity of visceral adipose tissue, which exerts a direct destructive effect on bone metabolism. Pro-inflammatory cytokines IL-6 and TNF-α, hypersecreted by visceral adipose tissue in obesity, stimulate osteoclast differentiation through activation of the RANK/RANKL/OPG pathway, enhancing bone resorption. Simultaneously, osteoblast function is suppressed, disrupting bone remodeling processes. Insulin resistance, characteristic of obesity, impairs osteoblast function by reducing type I collagen synthesis and the degree of bone matrix mineralization, which negatively affects bone quality [42,43]. It has been established that hyperinsulinemia in insulin resistance initially stimulates osteoblasts via insulin receptors, but chronic hyperinsulinemia leads to receptor desensitization and suppression of osteoblastogenesis [23,44]. Thus, the metabolic disturbances characteristic of obesity exert their negative effects on bone not only through inflammatory mechanisms but also through direct dysregulation of the endocrine function of adipose tissue. Adipose tissue influences bone metabolism through the secretion of adipokines. Leptin exerts a dual effect: peripherally, it stimulates osteoblastogenesis, whereas in obesity and the development of leptin resistance, it may reduce BMD. Reduced adiponectin levels in obesity are associated with impaired insulin sensitivity and enhanced systemic inflammation, which negatively affects bone quality [45,46,47]. Moreover, it is believed that obesity is generally accompanied by reduced physical activity due to limited mobility, joint pain, and general fatigue. The deficit of mechanical loading on the skeleton is an independent factor accelerating BMD loss [12,38]. A clinically significant consequence of OPO is the paradoxically increased risk of fractures despite an ostensibly protective high body weight.

**Figure 1:**
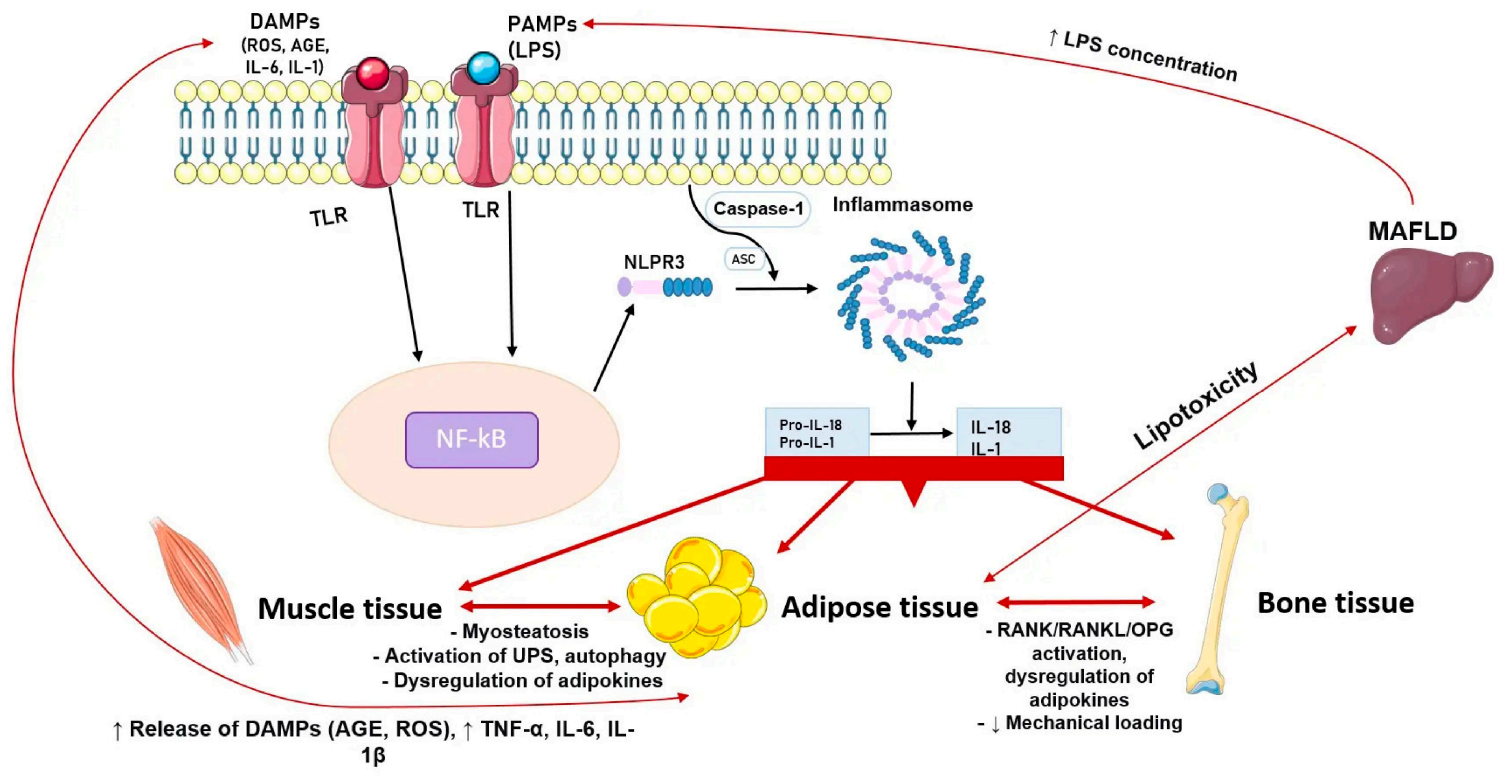
Common Pathogenetic Pathways of Chronic Low-Grade Inflammation Underlying the Formation of Body Composition Phenotypes. DAMPs—damage-associated molecular patterns; PAMPs—pathogen-associated molecular patterns; ROS—reactive oxygen species; AGE—advanced glycation end-products; LPS—lipopolysaccharide; TLR—Toll-like receptors; NLRP3—NOD-, LRR- and pyrin domain-containing protein 3; ASC—apoptosis-associated speck-like protein containing a caspase recruitment domain; NF-κB—nuclear factor kappa-B; IL-1—interleukin-1; IL-18—interleukin-18; TNF-α—tumor necrosis factor-alpha; UPS—ubiquitin-proteasome system; RANK—receptor activator of nuclear factor kappa-B; RANKL—receptor activator of nuclear factor kappa-B ligand; OPG—osteoprotegerin; MAFLD—metabolic dysfunction-associated fatty liver disease; ↑—increase, ↓—decrease in activity/function relative to the control/baseline. Image(s) provided by Servier Medical Art (https://smart.servier.com), licensed under CC BY 4.0 (https://creativecommons.org/licenses/by/4.0/) (accessed on 20 June 2026).

Metabolic dysfunction-associated fatty liver disease (MAFLD) is also associated with OPO through reduced hepatic IGF-1 synthesis in steatohepatitis, which impairs anabolic stimulation of bone tissue; insulin resistance suppresses insulin receptor signaling in osteoblasts; and vitamin D deficiency, characteristic of this nosology due to impaired hepatic 25-hydroxylation, further accelerates bone resorption. According to a systematic review and meta-analysis encompassing 19 studies (totaling over 133,000 participants), MAFLD is associated with reduced BMD and increases the risk of osteoporosis by 33% compared to individuals without MAFLD, with this association being most pronounced in populations with concurrent obesity and metabolic syndrome [48,49]. Thus, MAFLD represents one of the most clinically significant metabolic nosologies that consistently give rise to the OPO phenotype through convergence of hepatocellular dysfunction, insulin resistance, and systemic inflammation, Figure 1.

### 2.4. Osteosarcopenia

Osteosarcopenia (OS) is the combination of sarcopenia and osteopenia/osteoporosis in the same patient. The concept of OS is based on the notion of the bone–muscle unit, in which mechanical and biochemical interactions between these tissues are critically important for maintaining their functional integrity. Data on the prevalence of OS in the population vary widely, which is attributable to the heterogeneity of the diagnostic criteria employed and the characteristics of the study samples. Among elderly individuals aged over 65 years, the prevalence of OS ranges from 5% to 37% [50]. In the study by Huo Y.R. et al., which included 680 patients with a mean age of 80 years, approximately 37% had an osteosarcopenic state verified by DXA [51]. In this age group, OS significantly increases the risk of falls and fractures, hospitalization rates, the need for surgical interventions, and the risk of mortality, as well as reducing quality of life. At the same time, data on the prevalence of OS among younger individuals remain extremely limited: available studies are predominantly focused on the geriatric population, whereas targeted epidemiological investigations dedicated to the study of OS in young patients are virtually absent to date, which precludes reliable conclusions regarding the frequency of this condition in this age group.

#### Pathogenesis of Osteosarcopenia

The mechanical interaction between muscle and bone tissues is realized through Harold Frost’s mechanostat theory: muscle contractions generate stimuli that activate osteoblasts and suppress osteoclastogenesis [52]. Muscle tissue exerts paracrine effects on bone through myokines: irisin stimulates osteogenesis, IGF-1 promotes osteoblast proliferation, whereas myostatin acts as a negative regulator of both tissues, creating a vicious circle [53]. Common molecular mechanisms in OS include activation of NF-κB by pro-inflammatory cytokines, namely TNF-α, IL-6, and IL-1β. In muscle tissue, this leads to myocyte apoptosis; in bone, it results in enhanced RANKL-dependent osteoclastogenesis [54,55]. In bone tissue, cytokines increase RANKL expression on osteoblasts and stromal cells while simultaneously reducing the production of osteoprotegerin (OPG), a natural inhibitor of RANKL. As a result of the RANKL/OPG imbalance, pre-osteoclasts expressing the RANK receptor differentiate into mature osteoclasts and resorb the bone matrix. In parallel, IL-1β and TNF-α exert direct stimulatory effects on already mature osteoclasts, enhancing bone destruction. Alongside excessive resorption, the formation of new bone tissue is also suppressed: inflammatory cytokines inhibit the differentiation of mesenchymal stem cells (MSCs) into osteoblasts and activate caspase-mediated apoptosis of osteoblasts and osteocytes. A key anabolic signaling pathway suppressed in systemic inflammation is the Wnt/β-catenin pathway: its inhibition blocks osteoblast proliferation and mineralizing function, collectively creating a state of impaired bone remodeling—excessive resorption against a background of deficient bone formation [53]. Age-related deficiency of sex hormones and IGF-1 impairs anabolic signals in both tissues, accelerating their simultaneous degradation. Reduced muscle mass diminishes mechanical stimulation of bone, accelerates BMD loss, and fractures occurring against a background of osteoporosis cause immobilization and further exacerbate muscle atrophy.

Osteosarcopenia consistently develops in a number of pathological conditions that directly affect both bone and muscle tissue. Chronic kidney disease (CKD) is one of the most significant inducers of this phenotype, as mineral and bone disorder disrupts calcium, phosphate, parathyroid hormone, and FGF-23 homeostasis, leading to impaired mineralization of the bone matrix. Simultaneously, uremic metabolites, metabolic acidosis, and systemic inflammation induce anabolic resistance in muscle tissue [56]. From an epidemiological standpoint, the prevalence of osteopenia at the femoral neck in patients with CKD reaches 47% and 35% in the lumbar spine, while the global prevalence of sarcopenia in CKD is 24.5%, rising to 26.2% in patients on dialysis, and the fracture risk in dialysis patients exceeds the general population risk by more than 4-fold [57,58]. Thus, the vast majority of elderly patients with CKD have simultaneous involvement of both tissues, making osteosarcopenia a virtually obligatory complication of this nosology in its late stages.

### 2.5. Osteosarcopenic Obesity

Osteosarcopenic obesity (OSO) is the most complex of the phenotypes under consideration and represents the simultaneous combination of three pathological conditions: osteopenia/osteoporosis, sarcopenia, and obesity, meeting the diagnostic criteria for each. This phenotype was systematically described by Ilich J.Z. et al. in 2014 [5]. OSO is of greatest clinical interest, as the negative effects of the three constituent components are additive and mutually potentiating. Data on the prevalence of OSO are ambiguous due to the absence of recognized unified diagnostic criteria and a deficit of large epidemiological studies on this topic. In available studies, the prevalence of OSO varies substantially depending on age, sex, ethnicity, and the diagnostic criteria applied. According to a number of authors, prevalence may vary widely, ranging from 0.8% to 70.8%; among postmenopausal women, this figure ranges from 6% to 41% and increases with age [6]. The development of unified diagnostic algorithms is a priority direction for obtaining comparable population data.

At the same time, a fundamentally important epidemiological feature should be emphasized: in the vast majority of published studies, OSO is considered not as a specific complication of individual nosological entities, but as a natural age-associated condition, characteristic primarily of elderly and senile individuals. According to a systematic review and meta-analysis encompassing 21 studies with a total sample of 178,546 participants, significant independent risk factors for OSO include: female sex (OR 1.756; 95% CI 1.081–2.858), reduced physical activity (OR 1.562; 95% CI 1.127–2.165), and arterial hypertension (OR 1.482; 95% CI 1.207–1.821), whereas smoking, alcohol consumption, and dyslipidemia demonstrated no significant association [59]. Notably, it is age, sex, and lifestyle that emerge as the main determinants of OSO in the general population, reflecting its predominantly gerontological, rather than nosologically specific, nature. This circumstance does not diminish the significance of a number of clinical conditions, such as long-term glucocorticoid therapy, T2DM, and advanced-stage CKD, as conditions that accelerate the formation of OSO through specific molecular mechanisms and manifest it at a younger age [56,60].

At the same time, the marked heterogeneity of results between the studies included in the meta-analysis reflects a fundamental methodological problem of the entire field, namely the absence of unified diagnostic criteria for OSO, which directly accounts for the variability in prevalence estimates of body composition phenotype alterations. This limitation must be taken into account when interpreting the available epidemiological data.

Based on its pathogenesis, OSO represents a condition in which all the mechanisms described above converge: RANKL/RANK/OPG imbalance with reduced osteoblast function and osteoclast activation, suppression of the PI3K/Akt/mTOR pathway, persistent NLRP3 inflammasome activation with excessive IL-1β and IL-18 production, as well as pronounced dyslipidemia and lipotoxicity exerting damaging effects on muscle tissue (myosteatosis) and the liver (NAFLD). Particular attention should be paid to the role of glucocorticoids (GCs) as an iatrogenic factor in the formation of OSO. Long-term systemic GC therapy simultaneously suppresses osteoblastogenesis (glucocorticoid-induced osteoporosis), enhances muscle protein catabolism through GC receptor-dependent induction of atrogin, and promotes fat redistribution with visceral depot accumulation. Thus, patients with inflammatory and autoimmune diseases receiving long-term GC therapy represent a high-risk group for OSO and require mandatory screening of all body composition components [61].

**Figure 2:**
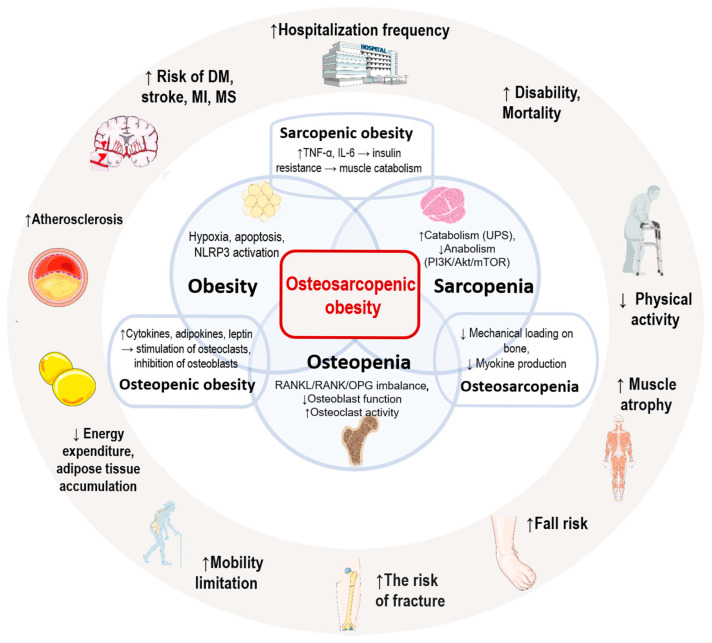
Pathogenesis of Combined Body Composition Phenotypes and Potential Complications. Note: TNF-α—tumor necrosis factor-alpha; IL-6—interleukin-6; UPS—ubiquitin-proteasome system; PI3K—phosphatidylinositol 3-kinase; Akt—protein kinase B; mTOR—mammalian target of rapamycin; NLRP3—NOD-, LRR- and pyrin domain-containing protein 3; RANKL—receptor activator of nuclear factor kappa-B ligand; RANK—receptor activator of nuclear factor kappa-B; OPG—osteoprotegerin; DM—diabetes mellitus; MS—metabolic syndrome; ↓—decrease in the parameter; ↑—increase in the parameter. Image(s) provided by Servier Medical Art (https://smart.servier.com), licensed under CC BY 4.0 (https://creativecommons.org/licenses/by/4.0/) (accessed on 20 June 2026).

It should also be noted that patients with OSO have a significantly higher risk of functional dependence, recurrent hospitalizations, and mortality compared to individuals with only one or two components of this condition [62,63]. The economic burden of OSO on healthcare systems is associated with the costs of fracture treatment, rehabilitation, long-term care, and management of comorbid metabolic diseases. Combined body composition phenotypes are associated with substantially more adverse clinical outcomes compared to isolated components, due to the synergism of pathological mechanisms, Figure 2. Individuals with SO have a 2- to 3-fold higher risk of disability compared to those with only sarcopenia or only obesity. An important aspect is the association of OSO with an additional burden on healthcare systems due to increased risk of falls, fractures, prolonged hospitalizations, and reduced quality of life, which determines the strategic importance of early screening [64].

### 2.6. From the Cross-Nosological Map of Phenotypes to Their Dynamics over Time

In all the nosological entities discussed above (T2DM, RA, COPD, CKD, MAFLD), the body composition phenotype is relatively stable and characterizes the disease as such, “fixing” the patient within a single phenotype that forms and intensifies as the disease progresses. However, there is a disease in which the same phenotypes do not coexist statically but rather successively replace one another in the same patient over time—type 1 diabetes mellitus. In its early stages, it manifests as catabolic depletion of adipose tissue, followed by excessive fat accumulation with normal or moderately elevated BMI (essentially normal-weight obesity), subsequently transforming into visceral sarcopenic obesity, and, in chronic decompensation, combined loss of muscle and bone tissue approaching the osteosarcopenic phenotype. Thus, T1DM transforms the “horizontal” cross-nosological map of phenotypes into a “vertical” trajectory of their sequential succession and serves as a convenient clinical model for studying the dynamics of body composition. The second part of this review is devoted to the examination of this trajectory. Unlike the cross-nosological phenotypes reviewed in Section 2.1, Section 2.2, Section 2.3, Section 2.4 and Section 2.5, which are each documented mainly through cross-sectional prevalence data in distinct disease populations, T1DM offers a within-patient, longitudinal illustration of how the same pathogenetic pathways described above (insulin/IGF-1 signaling, chronic low-grade inflammation, and glucocorticoid- or therapy-related effects on bone and muscle) can sequentially produce catabolic, normal-weight-obese, sarcopenic-obese, and osteosarcopenic phenotypes in a single individual over the course of the disease. This is the specific sense in which T1DM is used as a model in this review, and Part II is structured to follow this sequence explicitly.

## 3. Part II. Type 1 Diabetes Mellitus as a Dynamic Model of Sequential Body Composition Phenotype Shifts

Patients with T1DM are characterized by pronounced alterations in body composition, which significantly depend on the stage of the disease, duration of insulin therapy, and degree of metabolic control. In contemporary studies, these alterations are considered not as a static characteristic, but as a dynamic process that sequentially passes through several phases: a catabolic phenotype at disease onset, rapid weight gain upon initiation of insulin therapy, and the formation of an adiposity or sarcopenic phenotype in the setting of long-standing disease. Since BMI does not allow differentiation between fat and lean components, DXA, MRI, and BIA are increasingly employed for adequate assessment of these changes.

### 3.1. Body Composition at Disease Onset: The Catabolic Phenotype

The phenotype at T1DM onset is driven by absolute insulin deficiency, leading to activation of lipolysis, proteolysis, and caloric loss due to glucosuria [65]. At the molecular level, T1DM onset triggers the pathogenetic mechanisms described above as universal for catabolic body composition phenotypes. Under physiological conditions, insulin, via its receptor, activates the PI3K/Akt/mTOR signaling cascade, which ensures muscle protein synthesis and suppresses UPS-dependent proteolysis. In absolute insulin deficiency, this cascade is blocked: Akt phosphorylation is reduced, and the transcription factors FoxO1/3 are derepressed, activating the expression of atrogenes—the E3 ubiquitin ligases MAFbx/Atrogin-1 and MuRF-1, responsible for myosin and actin degradation via the proteasome. Simultaneously, suppression of autophagy, the second catabolic system of muscle proteolysis, is impaired: mTORC1 dephosphorylation releases inhibition of the ULK1 complex and triggers autophagic degradation of myofibrillar proteins. These mechanisms explain the loss of muscle mass at T1DM onset, although less pronounced than fat mass loss [66].

In parallel, insulin deficiency activates hormone-sensitive lipase (HSL) in adipocytes and adipose triglyceride lipase (ATGL), leading to uncontrolled lipolysis with release of free fatty acids (FFAs) and glycerol. The excess of circulating FFAs not only serves as a substrate for ketogenesis but also, upon accumulation in myocytes in the form of diacylglycerol and ceramides, activates protein kinase C—one of the key inducers of impaired insulin signaling in muscle tissue, which has been proposed as one biochemical contributor to skeletal muscle insulin resistance with increasing disease duration [66]; this pathway is derived largely from preclinical and mechanistic studies, and its quantitative contribution in patients with T1DM has not been directly established.

The most typical manifestation of T1DM at onset is weight loss, occurring predominantly through reduction in adipose tissue, whereas lean (fat-free) body mass decreases to a lesser extent or may remain relatively preserved. In the study by Rosenfalck A.M. et al. (2002) [67] using DXA in adult patients aged 31.5 ± 3.2 years with newly diagnosed T1DM, self-reported weight loss prior to treatment initiation averaged approximately 6 kg. During the following year after initiation of insulin therapy, body weight increased by an average of approximately 4.3 ± 2.9 kg (*p* = 0.0012), with fat mass increasing by approximately 13%, whereas lean body mass increased by 4.9%. In other words, weight gain in the early period of therapy is more than half accounted for by restoration and subsequent accumulation of adipose tissue [67]. Similar data were obtained in children. In the study by Davis N.L. et al. (2012) [68], children with T1DM at diagnosis had significantly lower BMI standard deviation scores compared to the control group: −0.67 (1.34) versus 0.20 (1.14), *p* < 0.05; a reduction in body fat percentage was also noted, namely 20.3% versus 24.5% in the control group (*p* < 0.05). In addition, it should be noted that girls with T1DM at diagnosis were leaner than boys, which was also observed in the study by Kibirige M. et al. (2003) [69], in which boys had significantly higher BMI standard deviation scores (0.56 versus −0.08 in girls, *p* = 0.006) and developed diabetes at a significantly earlier age (6.74 years versus 8.32 years in girls, *p* < 0.05); however, after adjustment for BMI, the age difference between sexes was no longer significant, which the authors interpreted as an association between T1DM onset and overweight or concomitant factors [68,69]. Subsequently, the “accelerator hypothesis” proposed by Terry Wilkin in 2001, which posited that weight gain leads to increased insulin resistance and impairs glycemic control, was not confirmed [70]. In the study by Kuchlbauer V. et al. (2014), the youngest children at diagnosis, conversely, had the lowest BMI standard deviation scores, and vice versa [71]. Furthermore, in the retrospective study by Islam S.T. et al. (2014) [72], it was found that over 15 years, the proportion of children with overweight and obesity at diagnosis of type 1 diabetes did not change significantly. This indicates that increased adiposity alone cannot explain the observed rise in the incidence of this disease in recent years [72].

### 3.2. Changes upon Initiation of Insulin Therapy: Transition to Normal-Weight Obesity

As mentioned above, a significant proportion of adult patients experience weight gain as early as the first year of therapy, with the increase occurring predominantly through adipose tissue. At the same time, glycemic control improves, reflecting the physiological restoration of insulin’s anabolic action following a period of decompensation [67]. In the study by Jacob A.N. et al. (2006) [73], in 21 patients with T1DM, body weight increased by 2.15 kg (2.69% of baseline) by the sixth month of follow-up. The main cause of weight gain is the lipogenic effect of insulin, leading to accumulation of fat mass [73]. The molecular basis of this imbalance is related to the differential sensitivity of adipose and muscle tissue to the restored insulin signal. Insulin activates the transcription factor SREBP-1c via the PI3K/Akt/mTORC1 cascade, inducing the expression of key de novo lipogenesis enzymes—acetyl-CoA carboxylase and fatty acid synthase—ensuring preferential energy deposition in adipose tissue [74]. Simultaneously, patients with T1DM in the early period of therapy maintain subclinical inflammation—a consequence of autoimmune inflammation and preceding decompensation—which sustains elevated levels of TNF-α and IL-6. These cytokines, via NF-κB, suppress transcription of myogenic regulatory factors and inhibit satellite cell differentiation, limiting muscle mass gain [66]. The result is precisely the imbalance described in Part I as defining normal-weight obesity: normal BMI with excess fat and relatively reduced muscle components. Increased physical activity may attenuate this process. The negative correlation of energy intake and appetite with fat mass gain suggests that these factors do not play a significant role in obesity, whereas energy intake is important for muscle mass gain, which was negatively correlated with weight gain [73].

In children and adolescents, body weight normalizes relatively quickly after initiation of insulin therapy. According to observations, within several weeks after starting treatment, fat and muscle mass parameters approach control values, and during the first year, significant differences from healthy peers are not detected in many cases [68,69]. The most intensive weight gain typically occurs during the first 1–3 months of therapy, particularly in patients who experienced diabetic ketoacidosis and had pronounced initial weight loss [65].

Since in the early period of therapy fat mass gain may exceed muscle mass gain by more than twofold, weight restoration should not be interpreted exclusively as a favorable sign. In some patients, so-called “excessive” (compensatory) adiposity develops—disproportionate accumulation of adipose tissue beyond simple restoration of the initial deficit. In essence, this condition corresponds to normal-weight obesity, described in Part I of the review as a hidden entry point into the spectrum of combined body composition phenotypes: the patient maintains a normal BMI with excess adipose tissue content. This phenomenon is most pronounced with high caloric intake, a tendency toward hypoglycemia requiring additional carbohydrate consumption, and intensive insulin regimens with relatively high total daily insulin doses [75].

### 3.3. Body Composition in Long-Standing Disease: Development of Sarcopenic Obesity

In long-standing T1DM, body composition characteristics are primarily determined by the quality of glycemic control and the degree of insulin resistance. In children and adolescents, most contemporary studies reveal increased total fat mass and body fat percentage compared to healthy peers. According to a meta-analysis of DXA studies, fat tissue content in children with T1DM is on average approximately 9% higher than in peers without diabetes, with no significant differences in lean mass. Moreover, earlier age at onset and higher total daily insulin dose are associated with greater differences in body fat percentage [65].

Despite normal or moderately elevated BMI, such patients frequently exhibit excess abdominal adipose tissue accumulation and signs of insulin resistance, which is regarded as a variant of “double diabetes” (combination of autoimmune T1DM with metabolic features typical of T2DM) [76]. In terms of body composition phenotype, this condition converges with sarcopenic obesity, the pathogenesis of which was discussed in Part I: visceral fat accumulation, insulin resistance, and chronic inflammation are accompanied by suppression of anabolic signals in muscle tissue. In T1DM, the implementation of these mechanisms has specific features related to the combination of exogenous hyperinsulinemia and chronic hyperglycemia. Accumulated AGEs in skeletal muscle bind to RAGE receptors, activating the NLRP3 inflammasome. Activated caspase-1 in myocytes induces processing of IL-1β and IL-18, amplifying the local pro-inflammatory cascade and accelerating degradation of sarcomeric proteins via the ubiquitin-proteasome system [27,66]. In parallel, AGEs impair mitochondrial function in myocytes: through excessive generation of reactive oxygen species, they suppress AMPK activity—a key sensor of cellular energy status, whose activation is required for maintenance of mitochondrial biogenesis and mTORC1-dependent anabolism. Thus, in “double diabetes,” the specific AGE/RAGE/NLRP3 pathway, driven by chronic hyperglycemia, is added to the universal mechanisms of SO [66]. Long-term intensive insulin therapy is associated with gradual weight gain and redistribution of adipose tissue predominantly to the abdominal type. These changes are associated with poorer glycemic control, dyslipidemia, and cardiovascular risk [75].

In adult patients with long-standing disease, a tendency toward increased fat mass with relative reduction in the proportion of lean body mass is also observed, with these changes being particularly pronounced in women. In a comparative study by Fenner-Pena N. et al. (2024) [77] using bioelectrical impedance analysis in adults with long-standing T1DM, a reduction in phase angle, reflecting cell membrane integrity and muscle tissue quality, was demonstrated compared to healthy controls, with women predominantly exhibiting increased adiposity and men showing reduced muscle mass. It was also noted that patients with T1DM have lower levels of physical activity, which may directly influence changes in body composition [77].

Of particular importance is the accumulation of visceral and abdominal adipose tissue. In patients with T1DM, increased visceral fat is associated with the development of insulin resistance, dyslipidemia, and a higher incidence of microvascular complications; central fat distribution (particularly increased trunk-to-peripheral fat ratio) is associated with the risk of diabetic neuropathy, including cardiac autonomic neuropathy, to a greater extent than overall BMI [75]. It has been established that poorer glycemic control and higher insulin doses are often accompanied by increased total and central fat mass [65].

**Figure 3:**
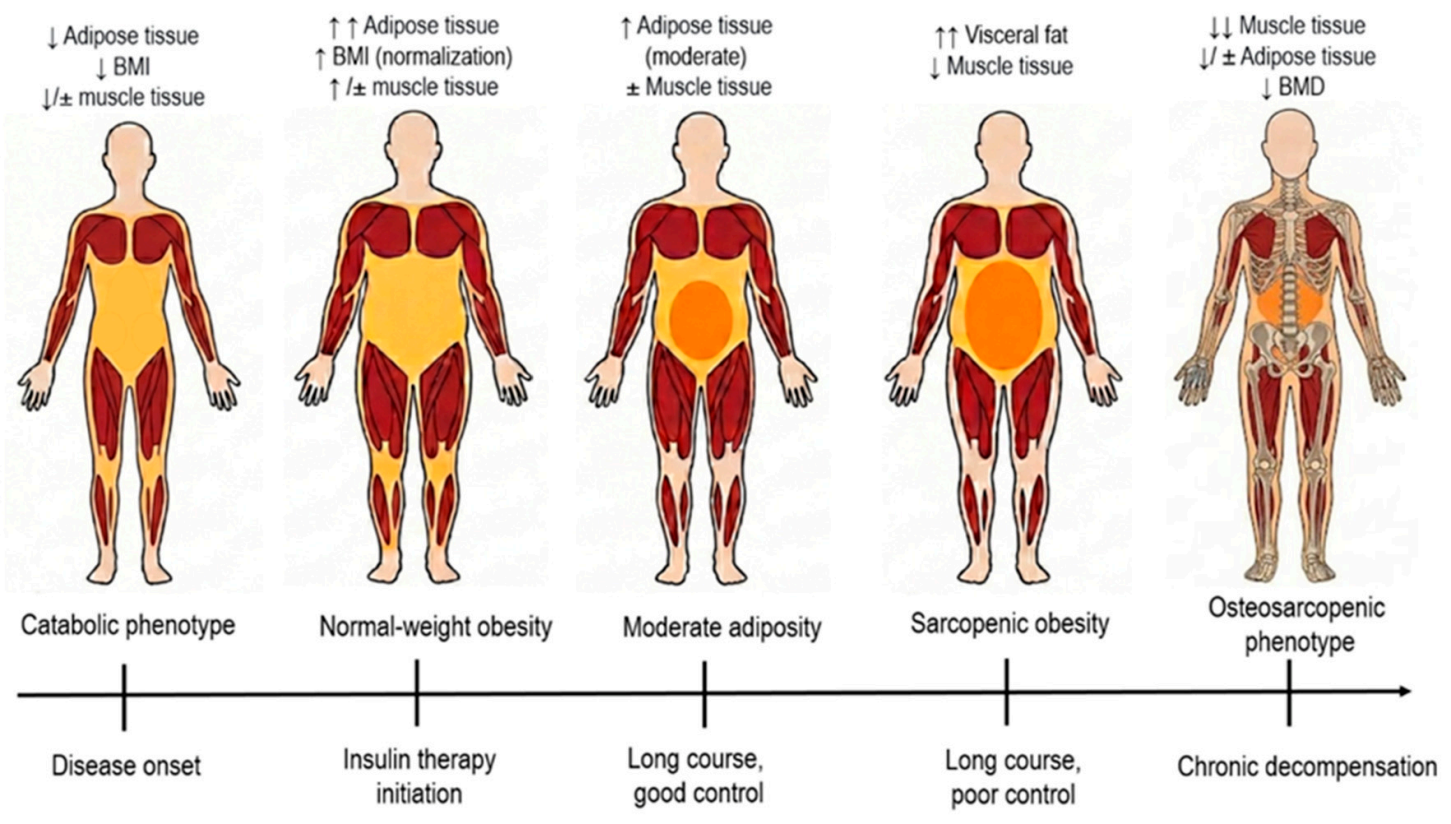
Changes in Body Composition in Type 1 Diabetes Mellitus. ↑, ↑↑, ↓, and ↓↓ indicate moderate increase, strong increase, moderate decrease, and strong decrease, respectively, in tissue amount (muscle, adipose, and bone); ±—parameter without significant changes; normalization—return of BMI to reference values; moderately—slight or moderate increase. BMI—body mass index (body weight divided by height squared, kg/m^2^); BMD—bone mineral density (measure of bone strength, assessed by densitometry); visceral fat—fat surrounding the internal organs (metabolically active). Generated using Google Gemini (Google, 2026).

The ratio of muscle to adipose tissue is of considerable importance. Regular physical activity is associated with reduced total and visceral fat mass, a more favorable metabolic profile, and better body composition parameters in patients with T1DM. In children and adolescents who engage in additional physical activity, bioelectrical impedance analysis phase angle values are comparable to those of healthy peers, whereas in their less active counterparts with T1DM, this parameter is reduced. It is noteworthy that children and adolescents with well-controlled T1DM demonstrated comparable body composition parameters [78]. A schematic representation of body composition changes in type 1 diabetes mellitus is presented in Figure 3.

### 3.4. Impact of Glycemic Control and Chronic Decompensation: Sarcopenic and Osteosarcopenic Shift

As mentioned above, the quality of glycemic control is one of the key factors determining the direction of body composition changes. On the one hand, intensification of insulin therapy and achievement of glycemic targets are naturally accompanied by restoration and gain of fat mass. On the other hand, chronic decompensation gives rise to a fundamentally different phenotype. Long-term hyperglycemia is accompanied by impaired protein metabolism, reduced muscle protein synthesis, accumulation of advanced glycation end-products in skeletal muscle, mitochondrial dysfunction, and chronic inflammation, which contribute to progressive reduction in muscle mass and the development of sarcopenic changes [79].

At the bone tissue level, chronic hyperglycemia recapitulates the mechanisms described in Part I with respect to osteosarcopenia. Reactive oxygen species and AGEs generated by hyperglycemia suppress the Wnt/β-catenin signaling pathway, inhibiting the expression of Wnt ligands and receptors while enhancing the expression of their antagonists, thereby suppressing bone formation and enhancing resorption [80]. AGEs accumulating in the collagen matrix of bone form cross-links in type I collagen molecules, disrupting their architecture and reducing biomechanical strength independently of mineral density. RAGE receptors on osteoblasts, upon binding with AGEs, activate NF-κB-dependent pro-inflammatory signaling, stimulating RANKL production while simultaneously reducing OPG secretion—via the same RANKL/OPG mechanism discussed in the osteosarcopenia section of Part I [81]. Elevated sclerostin levels, closely associated with hyperglycemia and AGE-mediated osteocyte dysfunction, further suppress Wnt/β-catenin activity, reducing osteoblastogenesis [82]. Since muscle atrophy progresses simultaneously, mechanical loading on the skeleton is also reduced—a stimulus for osteoblastogenesis according to Frost’s mechanostat theory—which further accelerates bone loss [66].

Notably, the accumulation of advanced glycation end-products (AGEs), mitochondrial dysfunction, and activation of inflammatory cascades represent the same universal mechanisms that were described in Part I of this review as the common pathogenetic basis of combined body composition phenotypes. The MUSCLES-DM study demonstrated that it is poor glycemic control, rather than diabetes type or insulin use, which constitutes the primary risk factor for sarcopenia. Moreover, in elderly patients with T1DM, the prevalence of sarcopenia and reduced handgrip strength was higher than in T2DM, indicating particular vulnerability of skeletal muscle in long-standing insulin-dependent disease [79]. In some patients with long-standing T1DM, a reduction in appendicular muscle mass and muscle strength is observed even with relatively stable total body weight and normal BMI. According to the study by Pollakova D. et al. (2023), the prevalence of reduced appendicular lean mass index in adults with long-standing T1DM reaches approximately 23.1% (25% in women and 21.74% in men), and the severity of muscle mass and strength reduction correlates positively with disease duration and inversely with physical activity level [83]. In the study by Andreo-López M.C. et al. (2023) [20], lower muscle mass and handgrip strength values were also associated with poorer glycemic control, underweight, and, moreover, with low adherence to the Mediterranean diet. However, there were more men in the sarcopenia and dynapenia groups [20].

In severe cases of chronic decompensation, re-formation of the catabolic phenotype with reduction in both fat and lean body mass is possible, effectively reproducing the metabolic picture of disease onset. Given that chronic hyperglycemia and AGE accumulation also affect bone tissue, long-term decompensated T1DM approaches the osteosarcopenic phenotype described in Part I in its profile. Thus, the impact of glycemic control on body composition is bidirectional: excessive fat mass gain with “liberal” control and intensive insulin therapy, on the one hand, and loss of muscle mass—and, with prolonged decompensation, bone mass—in chronic hyperglycemia, on the other.

### 3.5. Differences in Body Composition Across Different Modalities of Insulin Therapy

Accumulated data allow comparison of body composition in standard insulin therapy with multiple daily injections (MDI), insulin pump therapy (continuous subcutaneous insulin infusion, CSII), and the addition of glucagon-like peptide-1 receptor agonists (GLP-1 RAs) to insulin. From the perspective of body composition phenotype concept, the choice of therapy modality may be viewed as a tool for modifying the body composition phenotype.

#### 3.5.1. Standard Insulin Therapy (MDI)

Intensive insulin therapy in the multiple daily injection regimen is associated with a pronounced tendency toward gradual weight gain, particularly with high total daily insulin doses and intensification of glycemic control. According to the DCCT/EDIC study, weight gain with intensive therapy was significantly greater than with standard therapy, and its magnitude was primarily determined by the quality of glycemic control: greater annual weight gain was associated with lower achieved HbA1c levels, higher insulin doses, and a family history of type 2 diabetes mellitus [84].

#### 3.5.2. Continuous Subcutaneous Insulin Infusion (CSII)

Transition from MDI to pump therapy is accompanied by favorable changes in body composition. In a prospective study, 6 months after switching to CSII, patients of both sexes showed increased muscle mass, as well as a predominant reduction in visceral fat area in men and reduction in total fat mass in women. These changes were accompanied by reduced glycemic variability and increased physical activity levels [85]. At the same time, data on long-term weight dynamics are inconclusive. Although continuous subcutaneous insulin infusion provides better glycemic control and requires lower doses compared to multiple insulin injections, weight gain over a 10-year period was similar in both groups in the study by Alderisio A. et al. (2019) [86]. A longitudinal study of 35,624 children and adolescents from 2011 to 2016 revealed that adolescents, particularly girls using CSII, showed a trend toward higher body mass index [87].

Therapy with GLP-1 RA add-on. The addition of GLP-1 receptor agonists (in particular, liraglutide) to insulin therapy in overweight and obese adults with T1DM leads to weight loss predominantly through reduction in fat mass, including visceral fat, with preservation of lean body mass. In a randomized placebo-controlled trial using DXA and MRI, liraglutide improved glycemic control, reduced adiposity, and lowered systolic blood pressure; weight loss was accompanied by activation of lipid oxidation and thermogenesis mechanisms in adipose tissue with preservation of lean mass. In a separate randomized placebo-controlled trial in adults with T1DM, the magnitude of weight loss with liraglutide compared to placebo was 3.38 kg (95% CI −5.29; −1.48; *p* < 0.001) [88].

Similar effects have been described in patients receiving insulin pump therapy: the addition of liraglutide to CSII in overweight patients with poor glycemic control was accompanied by a reduction in hyperglycemia and mean body weight by 6.3 kg (*p* < 0.001) compared to placebo [89]. Thus, in contrast to intensification of insulin therapy per se, which leads to adipose tissue gain, the addition of GLP-1 RAs shifts body composition toward reduction in fat mass with preservation of the muscle component—that is, in the direction opposite to the formation of sarcopenic obesity. It is important to emphasize that the use of GLP-1 RAs in T1DM is currently not included in the registered indications and is considered as an additional (off-label) option, primarily in patients with obesity.

### 3.6. Principles of Management of Patients with Body Composition Disorders

Therapy for combined body composition phenotypes is aimed at simultaneous targeting of all three components through common pathogenetic targets. The key non-pharmacological intervention is structured physical activity. Resistance training significantly increases muscle mass and strength, stimulates osteoblastogenesis through mechanical loading, and promotes reduction in visceral fat. Moderate-intensity aerobic exercise additionally improves insulin sensitivity, while yoga or Pilates may improve balance and patients’ quality of life. A combination of all training types is optimal [90].

Nutritional correction in combined body composition phenotypes involves increased protein intake, predominantly from sources rich in leucine exceeding 2.5 g. Supplemental vitamin D and calcium are indicated when BMD is reduced. In SO and OSO, excessively low-calorie diets should be avoided due to the risk of accelerating muscle loss [91,92,93].

Among pharmacological approaches, GLP-1 receptor agonists, particularly semaglutide and tirzepatide, deserve special attention, as they provide reduction in visceral fat mass with relatively less loss of lean mass and improve glycemic control in T2DM, making them promising in SO and OSO in obese patients [94,95]. Antiresorptive therapy with denosumab and bisphosphonates is indicated when BMD is reduced. Notably, in aromatase inhibitor-associated OS, denosumab has demonstrated superiority over bisphosphonates in effects on trabecular bone index and body composition [96].

With respect to T1DM, the leading factor modifying the body composition phenotype remains insulin therapy itself: regimen selection (MDI or CSII), optimization of total daily dose, and quality of glycemic control directly influence the direction of changes in fat and muscle mass, as shown in Part II of this review. The addition of GLP-1 RAs in patients with obesity and T1DM, as well as regular physical activity, are considered additional tools for shifting the phenotype in a favorable direction.

Regarding the specific role of physical activity in T1DM, current guidance emphasizes both its cardiometabolic benefits and the distinct glycemic risks it entails. The ISPAD 2022 consensus guidelines recommend that children and adolescents with T1DM accumulate at least 60 min of moderate-to-vigorous physical activity daily, and highlight that aerobic exercise typically lowers glycemia during and after the session, whereas anaerobic and resistance exercise produce a smaller decline or even a transient rise in glycemia; exercise prescriptions therefore require individualized adjustment of insulin dosing and carbohydrate intake according to exercise type, intensity, and duration [97]. With respect to body composition specifically, a 2025 systematic review and meta-analysis of 25 randomized controlled trials in patients with T1DM (*n* = 1120) found that structured exercise training significantly reduced BMI, fasting glucose, and HbA1c and increased cardiorespiratory fitness (VO2max/peak) compared with non-exercising controls, but did not produce a statistically significant change in body fat percentage or lean body mass at the group level; age, exercise modality, and intervention duration were identified as the principal moderators of the observed effects [98]. These findings indicate that, while regular physical activity is warranted in T1DM for its cardiometabolic and functional benefits, its capacity to reverse or reshape body composition phenotypes specifically should not be overstated in the absence of larger, longer-duration trials stratified by age and exercise modality.

## 4. Conclusions

Combined body composition phenotypes represent a heterogeneous group of conditions united by common pathogenetic mechanisms, which serve both as manifestations of somatic diseases and as predictors of adverse clinical outcomes. Normal-weight obesity occupies a particular position within this spectrum, demonstrating the need for body composition screening using dual-energy X-ray absorptiometry or bioelectrical impedance analysis even in patients with normal body mass index. Current challenges in this field include the absence of internationally recognized unified diagnostic criteria for each of the combined phenotypes, a deficit of large prospective studies, insufficient data on prevalence across various population groups, and the lack of standardized therapeutic protocols. The integration of cross-nosological and dynamic perspectives on body composition phenotypes, including the example of T1DM, opens up the prospect of identifying common diagnostic approaches and therapeutic targets.

## 5. Limitations

This review is narrative rather than systematic in design: no formal reporting protocol (e.g., PRISMA), pre-registered search strategy, or quantitative pooling was applied, and study selection across the multiple disease areas and mechanistic pathways discussed was not exhaustive; consequently, selection bias affecting which studies were emphasized cannot be excluded. This article should therefore be read as a structured conceptual synthesis rather than as a systematic or quantitative evidence synthesis.

Several further limitations should be noted. First, much of the mechanistic synthesis presented in Part I and Part II—particularly the cytokine-, adipokine-, and intracellular signaling-based explanations linking systemic inflammation and insulin signaling to specific body composition phenotypes—is derived largely from preclinical, in vitro, or animal-model studies; direct longitudinal clinical confirmation in humans remains limited for several of these pathways, and this evidence should be interpreted as mechanistically plausible rather than clinically established. Second, diagnostic criteria for the combined phenotypes discussed (sarcopenic obesity, osteosarcopenia, osteopenic obesity, osteosarcopenic obesity) are not yet internationally standardized, and, as discussed in Part I, existing criteria for sarcopenia were developed primarily in older adults, limiting their direct applicability to the younger populations that are the focus of Part II; this heterogeneity constrains comparability of prevalence estimates across the studies cited. Third, the sequential trajectory of body composition phenotypes proposed for T1DM in Part II is synthesized from predominantly cross-sectional and short- to intermediate-term longitudinal studies conducted in different T1DM cohorts, rather than derived from a single prospective cohort followed through all described phenotypic stages; it should therefore be understood as an integrative model rather than as a directly demonstrated within-patient trajectory. Finally, evidence for several proposed therapeutic approaches (e.g., GLP-1 receptor agonists, structured exercise, antiresorptive therapy) in the specific context of T1DM-related body composition changes remains limited, and much of the supporting evidence is extrapolated from studies conducted in other populations, including individuals with type 2 diabetes mellitus, obesity, or postmenopausal osteoporosis.

## Data Availability

No new data were created or analyzed in this study. Data sharing is not applicable to this article.

## Abbreviations

The following abbreviations are used in this manuscript:

BC	Body composition
FM	Fat mass
BMI	Body mass index
LM	Lean mass
BMD	Bone mineral density
CT	Computed tomography
DXA	Dual-energy X-ray absorptiometry
T1DM	Type 1 diabetes mellitus
NWO	Normal-weight obesity
SO	Sarcopenic obesity
BIA	bioelectrical impedance analysis
T2DM	Type 2 diabetes mellitus
OPO	Osteopenic obesity
MAFLD	Metabolic dysfunction-associated fatty liver disease
OS	Osteosarcopenia
CKD	Chronic kidney disease
OSO	Osteosarcopenic obesity
MDI	Multiple daily injections
CSII	Continuous subcutaneous insulin infusion
GLP-1	Glucagon-like peptide-1 receptor agonists
